# Krüppel-Like Factors 4 and 5: Unity in Diversity

**DOI:** 10.2174/138920209789503932

**Published:** 2009-12

**Authors:** Inderpreet Sur

**Affiliations:** Department of Biosciences and Nutrition, Karolinska Institutet, SE 141 57 Huddinge, Sweden

## Abstract

Krüppel-like factors (Klf) 4 and 5 belong to a family of zinc finger-containing transcription factors that share homology with the Drosophila gene Krüppel. They regulate proliferation and differentiation of a wide variety of cells and have been linked to tumorigenesis. Their most striking role so far has turned out to be their ability to reprogram/ maintain embryonic stem cell fate. In this review, the data available in the field regarding their role in proliferation and differentiation and their coupling to carcinogenesis are summarized. The emphasis is on their context dependence and how they might be able to regulate diverse transcriptional outputs from the genome.

## 1. KRÜPPEL-LIKE FACTORS

Krüppel-like factors (KLFs) are zinc finger proteins that belong to the Sp1/Klf family of transcription factors. This family is characterised by a highly conserved C-terminal DNA-binding domain containing three zinc fingers which are similar to those found in the drosophila protein Krüppel. In drosophila, the amorphic mutation of Krüppel causes the lack of all three thoracic segments along with five of the eight abdominal segments. The missing abdominal segments are partially replaced by the mirror-image duplication of the normal posterior-abdominal segments [1]. This gives the embryo a crippled look, hence the name for the family. At least 17 Krüppel like factors belonging to this family, have so far been identified in mammals. These play diverse roles during differentiation and development [2-4].

### 1.1. Klf4 and Klf5

Klf4 and Klf5 are two of the family members that often show overlapping as well as mutually exclusive expression patterns. For instance, Klf4 is expressed prominently in the differentiating compartment of the gut [5]. Klf5 expression on the other hand is associated with the crypts of intestinal villi, which are active sites of proliferation [6]. Similar to the pattern in the intestine, Klf4 is expressed in the differentiating cells of the epidermis [7] while Klf5 is enriched in the basal layer or the proliferating compartment of the epidermis [8]. A detailed comparison of the expression pattern of these genes reveals interesting temporal differences during development. For instance, in the embryonic day 10.5 (E10.5) mouse embryo, Ohnishi *et al.* [8] did not detect any Klf4 expression in the primitive gut, which instead had high level expression of Klf5. At E15.5, Klf4 and Klf5 transcripts were similarly abundant in the developing gut. As development progressed, at E17.5, Klf4 transcripts were nearly absent in the intestine while the Klf5 expression remained still high in the intestine. A temporal regulation of Klf5 during development was also detected by Conkright *et al.* [6]. In this study high level expression of Klf5 was observed in E7 embryos, it was completely absent at day 11 and low levels of expression returned at day 15 and 17.

In the adult mice, Klf4 expression is high in the colon (proximal and distal) [5]. Moderate levels of transcript are also detected in the distal small intestine, testis and lung. In this study, no appreciable amount of message was detected in the brain, kidney, liver, spleen, thymus, heart, muscle and fat. Further, *in situ* hybridization showed that Klf4 was primarily expressed from the middle to upper region of the colonic crypt epithelium, indicating that it is expressed in cells that are in the process of migrating from the base towards the top of the crypt. Klf5 in the adult mice is predominantly expressed in the stomach, small intestine and colon. In addition lower levels of transcript are detected in the skin, lung, uterus, placenta and testis [6]. A comparison of the expression pattern in human and mice shows that in both these species, Klf5 is enriched in the small intestine and colon [6, 9]. Although identical tissues were not represented on the Northern blots in the two studies, some differences between mouse and human expression pattern could still be inferred, for instance high level expression of Klf5 was observed in the skeletal muscle in humans, while its expression was very weak in the case of mice. We have also looked at its expression in the human skin and found that it is highly expressed in the epidermis especially in cells of the inner root sheath and matrix of the hair follicles [10].

## 2. ROLE IN PROLIFERATION

From the expression pattern of Klf4 and Klf5 it was inferred that Klf5, being expressed in the proliferative compartment of both the skin and intestine might function as a pro-proliferative factor while Klf4 which is expressed in the differentiating compartment has growth inhibitory functions. Results from several laboratories have corroborated this idea. In one of the earliest reports on Klf4, transfection of Klf4 into NIH3T3 fibroblasts, inhibited their DNA synthesis. Further, its expression was induced in growth inhibitory conditions like serum starvation and contact inhibition [5]. Subsequently the ability of Klf4 to inhibit proliferation has been demonstrated in additional settings. For instance, it inhibits the proliferation of vascular smooth muscle cells *via* the induction of p53 [11]. In B-cells, Klf4 regulates cell number and activation-induced B-cell proliferation [12, 13]. Stimulation of proliferation of mature B-cells from mouse spleen with anti-IgM antibodies *in vitro* resulted in a rapid downregulation of Klf4. As expected, the forced expression of Klf4 in proliferating B-cells caused an arrest or delay in cell cycle progression. These cells consistently had an increased percentage of cells in the G1 phase of the cell cycle and a decreased percentage in the S phase [12]. Thus Klf4 acts as a quiescent factor for B cells. A similar function has been attributed to Klf4 in T-cells [14]. Ectopic expression of Klf4 in pancreatic cancer lines also leads to cell cycle arrest and marked growth inhibition *in vitro* [15]. It has been further shown that Klf4 can inhibit cell proliferation by blocking the G1/S progression of the cell cycle [16] and that Klf4 can directly repress *CyclinD1* promoter [17].

Klf5 on the other hand is pro-proliferative in several settings. Constitutive expression of Klf5 in NIH3T3 cells causes accelerated cell growth [18]. Further, these cells exhibit serum and anchorage independent growth. In this study, overexpression of Klf5 was sufficient to cause a transformed phenotype as assessed by focus formation and loss of contact inhibition. The pro-proliferative effect of Klf5 in non-transformed cell lines from the intestine and skin has also been demonstrated [19-21]. Additionally, the growth inhibition of IEC6 (a non transformed intestinal epithelial cell line) by all-trans retinoic acid (ATRA) can be overcome *via* Klf5 overexpression [22]. This corresponds well with the downregulation of Klf5 seen upon ATRA treatment in this cell line.

Although, as described above, Klf4 functions as a growth inhibitor and Klf5 as a pro-proliferative factor, both factors surprisingly exhibit context dependent effects. For Klf4, it is best exemplified by the study of Rowland *et al.* [23], in which Klf4 overexpression in wildtype mouse embryonic fibroblasts (MEFs) resulted in a tight proliferative arrest within 2 days. In contrast, in MEFs carrying an oncogenic RAS^v12^, Klf4 overexpression bypassed the Ras^v12^ induced senescence. Its expression in these cells led to cellular proliferation, loss of contact inhibition, an ability to grow in an anchorage-independent manner and resistance to cisplatin (a commonly used anti-cancer drug)-induced apoptosis. Mechanistically, Klf4 simultaneously repressed p53 (which promotes cell proliferation) and induced p21^cip1^ (which inhibits cell proliferation) expression. In normal cells, the p21^cip1^ induction by Klf4 represents the dominant response, resulting in cell-cycle arrest. In Ras^v12^ expressing cells, the increased cyclin-D1 levels neutralize the p21^cip1^ function. This leaves the inhibition of p53 by Klf4 as the dominant function, thereby, promoting cell proliferation and/or cell survival. Thus Klf4 although acting as an inhibitor of proliferation in many settings, possesses a pro-mitogenic activity that is uncovered only within a specific genetic context. At this point it is not clear how Klf4 simultaneously activates the p21 promoter and represses the p53 promoter. When it comes to Klf5, the scenario is a bit more clearer.

Similar to Klf4, Klf5 also shows context dependent roles. Although it is known to promote proliferation it can also be anti-proliferative. For instance, in primary intestinal cells, overexpression of Klf5 generally leads to an increased proliferation. Its overexpression in transformed cells however, causes growth arrest [19]. A similar outcome has been observed upon overexpression of Klf5 in several cell lines [24-27]. The context dependent function of Klf5 in regulating proliferation is further illustrated by the observation that Klf5 was required for proliferation of the spontaneously immortalized keratinocyte cell line HaCat. Surprisingly, it was also required for TGFβ-mediated growth inhibition. In this study, knockdown of Klf5 in HaCat cells resulted in an inhibition of cell proliferation. Although these cells exhibited reduced proliferation compared to the control, they failed to undergo growth inhibition upon TGFβ stimulation [21]. The authors further showed that acetylation of Klf5 is responsible for the TGFβ-mediated functional reversal of Klf5. The TGFβ-mediated acetylation of Klf5 altered its protein interactions and promoter binding, thereby, switching it from a transcriptional activator to a repressor on the p15(CDKN2B) promoter [26].

## 3. ROLE IN DIFFERENTIATION

Several mouse models have been generated which either lack the expression or ectopically express these factors. These models have clearly defined the roles of Klf4 and Klf5, not only in regulation of proliferation but also in regulating differentiation programmes of cells. A summary of phenotypes arising due to alterations in the Klf4 or Klf5 activity in mice is provided in Fig. (**1**).

### 3.1. Klf4

Knockout (KO) of Klf4 in mice results in neonatal lethality [7]. The *Klf4^-/-^* mice were born at the expected mendelian ratio and were initially indistinguishable from their littermates but died within 15 hrs of birth. Further examination revealed that the neonatal lethality was due to a defective skin barrier formation resulting in rapid dehydration and death. The importance of Klf4 in generating the skin barrier was further demonstrated in a reciprocal experiment where ectopic expression of Klf4 in the basal layer, *via* the keratin5 promoter resulted in an accelerated barrier acquisition [28]. While in control mice the barrier formation initiates at E16.5 and is fully established by E17.5 during development, the K5-*Klf4* bitransgenic animals had almost completely established the barrier by E16.5. At both E16.5 and E17.5, these mice also exhibited an approx 40% decrease in proliferation in the skin. The differentiation pattern was not altered. Interestingly, the Klf4-mediated acceleration of skin barrier formation is similar to the increased barrier acquisition in human fetus upon antenatal administration of corticosterone. Transcriptional profiling of dorsal skin from *Klf4^-/-^*, K5-*Klf4* and Dexamethasone (a corticosteroid)-treated mice further showed that Klf4 and corticosteroids target overlapping set of genes [29].

In another study, postnatal overexpression of Klf4 in the basal layer of the epidermis *via* the Keratin14 promoter led to rapid development of hyperkeratosis, atrophy of the sebaceous glands, cystic degeneration of hair follicles and hyperplasia [30]. These changes developed by 9 days of transgene induction. The *K14-rtTA;TRE-Klf4* mice further developed dysplastic changes in the skin by 3-4 weeks of induction. These dysplastic changes progressed into SCC *in situ*. The Klf4-induced dysplasia could be promoted by p53 deficiency and the authors further showed that the transforming ability of Klf4 required its nuclear translocation. At present a direct comparison of K5-*Klf4* and *K14-rtTA;TRE-Klf4* mouse models is not available. Since Jaubert *et al.* [28] used the mouse allele while Foster *et al.* [30] used the human allele of Klf4, it would be interesting to determine if there are any species-specific differences.

Klf4 deficiency also results in abnormalities in the colon. Since the *Klf4^-/-^* animals die within 15 hours of birth, these animals were examined on postnatal day1, the latest stage of intestinal differentiation before death [31]. Although the cell proliferation and cell death rates were normal in these animals, the colonic mucosa appeared abnormal and there was a dramatic reduction of mature goblet cells (by 90%). These are specialized epithelial cells that protect the intestinal mucosa from injury and help in repair. The colonocytes and enteroendocrine cells, the other specialized cells of the colon were not altered. Loss of goblet cells is also observed in mice with a conditional deletion of Klf4 in the eye *via* Le-Cre mediated recombination [32]. While at postnatal day 1 *Le-Cre/Klf4 ^loxP/loxP ^*mice look normal, by 8 weeks they develop multiple ocular defects, including corneal epithelial fragility, stromal edema, defective lens and loss of conjunctival goblet cells. Interestingly, the reduced number of epithelial cell layers observed in these mice was not due to reduced rate of cell proliferation but rather due to increased sloughing/fragility of the epithelium. In order to obtain mechanistic insight into the diverse ocular surface phenotype of these mice, the group further compared the gene expression patterns between the WT and *Le-Cre/Klf4 ^loxP/loxP ^*mice and found that Klf4 contributes to corneal homeostasis by coordinately regulating the expression of subsets of genes involved in specific functions such as cell cycle progression, cell-cell adhesion, epithelial barrier formation, expression of corneal crystallins and maintenance of corneal hydration [33].

Conditional ablation of Klf4 in the stomach *via* Foxa3-Cre results in gastric hypertrophy and mucus cell hyperplasia, starting at 2 weeks of age. The *Foxa3-Cre/Klf4^loxP/loxP^* mice had a 4-fold increased proliferation by 6 months of age. The adult gastric unit consists of at least 5 different mature cell types: the pit or surface mucous cells, which produce mucins and other factors involved in mucosal protection; the parietal or oxyntic cells, which secrete acid; the zymogenic or chief cells, which secrete pepsin; the enteroendocrine cells, which provide a number of gastric hormones including gastrin; and the caveolated or brush cells. Klf4 deletion resulted in a 2-fold increase in the number of pit cells and a 4-fold increase in the number of mucus neck cells. The number of parietal and zymogenic cells on the other hand was reduced to half. The enteroendocrine cells per gland and the apoptotic rate was not altered. From this study, it was concluded that Klf4 is required for directing the cell-fate decisions of the gastric epithelial precursor cells [34].

Conditional ablation of Klf4 in Sertoli cells of the testis did not affect proliferation. In the *AMH-Cre/Klf4^loxP/loxP^* mutant mice, the seminiferous tubules exhibited a disorganised germinal epithelium. Amongst the genes whose expression was altered in these mice, the authors found a number of genes involved in the maintenance of the differentiation function and/or directed vesicle transport in polarized epithelial cells [35].

Klf4 is also expressed in a monocyte-restricted and stage-specific pattern during myelopoiesis and functions to promote monocyte differentiation [36]. Hematopoetic stem cells (HSCs) generate committed progenitor cells that loose the capacity to self renew and ultimately differentiate along a specific lineage to form mature blood cells. In the myeloid pathway, mature monocytes and granulocytes arise from bipotential granulocyte/macrophage progenitors (GMPs) that, in turn, arise from multipotential common myeloid progenitors (CMPs); CMPs may also give rise to bipotential megakaryocyte/erythrocyte progenitors (MEPs). Overexpression of Klf4 in promyelocytic HL-60 cells or in primary CMPs or HSCs from bone marrow, restricts these cells along the monocyte/macrophage lineage at the expense of other myeloid lineages and confers the morphologic, genetic, and functional characteristics of a mature monocyte. Reciprocally CMPs derived from wildtype or *Klf4^loxP/loxP^* mice were retrovirally transfected with Cre recombinase and subsequently grown in medium capable of differentiating the cells along all myeloid pathways. CMPs with Klf4 deletion showed marked reduction of upto 56% in the formation of mature monocytes, whereas granulocyte formation was increased by 36%. Similarly Cre-excision of Klf4 in HSCs from *Klf4^loxP/loxP^* resulted in a 40% reduction in the number of monocytes and a 41% increase in granulocyte formation. These results thus show that varying amounts of Klf4 may dynamically regulate the balance between monocytes and granulocytes.

### 3.2. Klf5

Mice lacking Klf5 die during embryogenesis [37, 38]. The heterozygous mice survive and their analysis has defined several functions of Klf5. The *Klf5^+/-^* mice show reduced responses to injury and angiogenesis [37]. In response to external stress these mice had diminished arterial wall thickening, angiogenesis, cardiac hypertrophy and interstitial fibrosis. The activation and proliferation of smooth muscle cells and fibroblasts in response to vascular injury was impaired. In this model, Klf5 was identified as one of the transcription factors mediating Angiotensin II induced cardiovascular remodeling which could explain parts of the observed phenotype. It was also shown that Klf5 increased the expressions of platelet-derived growth factor-A (PDGF-A) and TGF-β during vascular remodeling and that this activity of Klf5 could be modulated by retinoic-acid receptor (RAR) ligands.

The *Klf5^+/-^* mice also exhibit defects in adipocyte differentiation [39]. The neonatal *Klf5^+/-^* mice have reduced mass of white adipose tissue (WAT). There was no difference in the number of adipocytes between the *Klf5^+/-^* and wildtype mice. However, a number of cells were small and contained little or no lipid droplets which normally are produced upon adipocyte differentiation. Although clearly reduced in neonates, by 4 weeks of age the WAT mass and morphology was comparable to the wild type. The development of brown adipose tissue (BAT) was not affected. In the *in vitro* model of adipocyte differentiation, introduction of a dominant negative Klf5 construct into the preadipocytes 3T3-L1 cells resulted in an almost complete block of the formation of lipid droplets. Conversely overexpression of Klf5 in these cells led to their spontaneous differentiation in confluent cultures. Mechanistically, Klf5 acted in concert with C/EBPβ and δ to activate the *PPARγ_2_* promoter that in turn controls a number of adipocyte-specific genes. The *Klf5^+/-^* mice also have skeletal growth retardation in the perinatal period. Although chondrocyte proliferation and differentiation were normal, cartilage matrix degradation was impaired in these mice causing a delay in replacement of cartilage with bone at the primary ossification center in the embryonic limbs. This defect was attributed to the effect of Klf5 on matrix metalloproteinase 9 (MMP9) expression which is required for matrix degradation, calcification and vascularization of the skeleton [40].

Although the *Klf5^+/-^* mouse model clearly demonstrates the effects of Klf5 on non-epithelial cells, a detailed histological picture of the effect of Klf5 deletion on the colon/ skin epithelial cells is currently not available. We have overexpressed Klf5 in the basal layer of the epidermis and found that its overexpression leads to loss of proliferation and misexpression of differentiation markers in the epidermis during development [41]. The overexpression of Klf5 also results in the disruption of epithelial-mesenchymal crosstalk causing defects in the underlying skeleton. This is quite striking since the overexpression of Klf5 is restricted only to the epidermis. When we induced Klf5 expression in adult mice, localized proliferation and epidermal erosions were observed. This phenotype was associated with a loss of the stem cells of the hair follicle bulge. These results demonstrate that 1) Klf5 can regulate differentiation states of the keratinocyte and 2) its effect on proliferation in the epidermis is context-dependent. Additionally even though the differentiation pattern was altered in this model, Klf5 overexpression did not lead to expansion of any one particular lineage. Based on these data we proposed a model wherein Klf5 expression is detrimental for cells capable of lineage commitment while it might be pro-proliferative in lineage committed cells of the epidermis. This idea is presently under investigation. It would be very interesting to determine as to what will be the outcome of Klf5 deficiency in the epidermis. Although the *Keratin5* promoter is also expressed in the esophagus resulting in Klf5 overexpression there as well, we did not detect any gross abnormalities. This is consistent with results of Goldstein *et al.* [42] who have overexpressed Klf5 throughout the esophageal epithelia *via* the Epstein-Barr virus (EBV) *ED-L2* promoter. They however, did observe increased proliferation in the basal layer. The number of proliferating cells was increased two-fold at both 1 mo and 1 yr of age. However, the suprabasal layers undergoing differentiation did not show increased proliferation despite the ectopic expression of Klf5.

Recently *Klf5^loxP/loxP^* mice have been generated which allows for conditional deletion of Klf5 in various tissues [43]. So far these mice have been bred into (TetO)7-Cre^-/tg^ and SFTPC-rtTA^-/tg^ mouse strains. In these mice, Klf5 was deleted in the respiratory epithelial cells of the triple transgenic mice upon doxycycline treatment [43]. Deletion of Klf5 during embryonic lung development resulted in the death of *Klf5^∆/∆^* mice shortly after birth due to respiratory distress. The authors found no effect of Klf5 deletion on proliferation of pulmonary epithelial or mesenchymal cells in the lungs of E15.5 embryos. Instead, the deletion of Klf5 in the respiratory epithelial cells inhibited morphological and biological maturation of the lung. Klf5 was found to regulate the maturation of both typeI and typeII epithelial cells. Klf5 deficiency in the respiratory epithelium also altered the underlying mesenchyme. Expression of αSMA, a marker of bronchiolar and vascular smooth muscle cell differentiation, was markedly increased in the bronchioles of the lungs from *Klf5^∆/∆^* mice. Additionally the normal association of epithelial and endothelial cells was disrupted. Microarray analysis identified alterations in several genes linked to paracrine signaling *via* TGFβ, PDGF, Vascular endothelial growth factor (VEGF) and Fibroblast growth factors (FGF).

## 4. ROLE IN STEM CELLS

Apart from the proliferation and differentiation functions of Klf4 and Klf5 discussed in the preceding sections, the most striking role for this family was recently demonstrated when it was found that Klf4 together with additional factors (Oct4, Sox2 and c-Myc) has the capacity to reprogram fetal as well as adult fibroblasts into pluripotent embryonic stem cells [44-47]. Further it has been established that a core Klf circuitry of Klf2, Klf4 and Klf5 regulates self-renewal of embryonic stem cells (ESC) [48]. This is the best evidence to date that Klf4 and Klf5 can perform similar function. This could also explain why *Klf4^-/-^* mice are viable but die soon after birth due to barrier defects. Other family members might compensate for Klf4 loss during development. Klf5 on the other hand appears to be indispensable for derivation of ESC from inner cell mass (ICM) of the mouse embryo [38]. In this study Ema *et al.* showed that Knockout (KO) of Klf5 resulted in an implantation defect of the embryos due to their inability in generating a functional trophoectoderm. They further showed, in an outgrowth assay, that the majority of *Klf5*^-/-^ blastocysts failed to attach to the dish and ESCs could not be derived from *Klf5^-/-^* inner cell mass. The group instead established the *Klf5^-/-^* ESC cell lines by replacing the wt allele in the *Klf5^+/-^* ESC with an IRES-hyg^r^ cassette. Early passage *Klf5*^-/-^ ESCs, appeared normal but later passage cells exhibited a differentiated morphology. Although these cells maintain pluripotency at the molecular level as determined by the unaltered expression of *Oct3/4*, *Rex1* and *Nanog*, the differentiation markers like *Fgf5*, *Brachyury* and *Cdx2* were increased in *Klf5^-/-^* ESCs. These cells when provided with an environment permissive for differentiation could differentiate earlier than the wildtype cells. Thus KO of *Klf5* results in priming of these cells for differentiation. In this regard it is significant that triple knockout of Klf2, Klf4 and Klf5 results in a gene expression signature which is similar to EpiSC, a pluripotent cell line derived from the epiblasts [48]. The EpiSC are pluripotent, but their pluripotency is more restricted than that of embryonic stem cells. Interestingly, the differentiation defect of *Klf5^-/-^* ESCs can be compensated by Klf4. On the other hand *Klf5^-/-^* ESCs also have a defect in proliferation which cannot be compensated by Klf4 [38]. These data suggest that the proliferation and differentiation are independent functional arms of Klf5. It also needs to be mentioned that contrary to the above results, Jiang *et al.* [48] in their study did not find any significant self renewal defects due to knockdown of Klf5 suggesting that a complete knockout of Klf5 is required.

Although Klf5 is essential for pluripotency, it is downregulated in several adult stem cells. As discussed before, in the epidermis Klf5 is expressed in the basal layer that contains the proliferating compartment while Klf4 is expressed in the suprabasal layer and regulates the expression of genes required for the formation of the barrier. The hair follicle contains a stem cell niche defined by the bulge region. Transcriptional profiling of the bulge cells isolated by the Krt1-15 expression and their comparison to the non-bulge basal keratinocytes showed that Klf5 was downregulated (five fold) in the stem cell compartment [49]. Similar results were obtained in transgenic mice expressing Tcf3 [50]. Tcf3 governs stem cell features and represses cell fate determination in the skin. Klf5 was downregulated in the developing epidermis at time points correlating with Tcf3 expression. Further, overexpression of Tcf3 in the epidermis led to an expression of the bulge stem cell transcriptional profile. This expression profile was also associated with a downregulation of Klf5. Finally it was shown that Tcf3 actively bound the Klf5 promoter in this study. Klf5 is also downregulated in Lgr5+ stem cells of the hair follicle [51]. The Lgr5+ cells constitute an actively cycling stem cell population of the hair follicle. The data from both the Lgr5 and Tcf3 study further suggests that the downregulation of Klf5 in the adult stem cells of the hair follicle is not linked to quiescence. Additionally Klf5 downregulation in these cells does not appear to be linked to stemness *per se* since hair germ cells that arise from the bulge cells show increased expression of Klf5 inspite of retaining several but not all features of stemness [52].

The downregulation of Klf5 observed in adult epidermal stem cells is not limited to the epidermis alone. A genetic and functional comparison of neural stem cells and embryonic stem cells showed a downregulation of Klf5 in the neural stem cells. In this study, Klf4 was also downregulated but to a more modest extent [53]. Thus from these data it appears that Klf4/ Klf5 might function more as factors whose presence or absence restricts fates. Since Klf4 and Klf5 are transcription factors that can bind DNA and alter target gene expression, it is possible that by regulating the assembly of signal-induced transcription factors on specific sites on the genome, they might influence diverse fates. 

## 5. TRANSCRIPTIONAL REGULATION

Although in Drosophila, Krüppel functions primarily as a transcriptional repressor, in mammals, Krüppel-like factors can function as transcriptional activators or as transcriptional repressors. Several stimuli are known to activate Klf4 [54-63] and Klf5 [20, 22, 64-71]. The activation of these factors regulate a variety of target genes belonging to diverse cellular processes [72, 73]. How these proteins accomplish such diverse functions or how the specificity is generated is still an open question. Protein modifications which may alter not only their transactivation functions but also their interactions with other proteins are likely to play a major role. These factors are known to be ubiquitinated [74-77], phosphorylated [78], acetylated [21, 26, 79, 80] and SUMOylated [81-83]. How protein modifications alter their function is best exemplified by the observation that SUMOylation functions as a molecular switch in converting Klf5 from a repressor to a transactivator in transcriptional programs of lipid metabolism involving Peroxisome proliferator-activated receptor-δ (PPAR-δ) [82]. In this study, it was elegantly shown that Klf5 modified with small ubiquitin-related modifier (SUMO) proteins was associated with transcriptionally repressive regulatory complexes containing unliganded PPAR-δ and co-repressors NCor, SMRT. In this state, Klf5 inhibited the expression of *Cpt1b*, *Ucp2* and *Ucp3* which are genes involved in lipid oxidation and energy uncoupling. Upon agonist stimulation of PPAR-δ, Klf5 was deSUMOylated and became associated with transcriptional activation complexes containing both the liganded PPAR-δ and CREB binding protein (CBP). Similar to the effect of SUMOylation described above, acetylation of Klf5 can also reverse its function [21, 26]. It was shown that knockdown of Klf5 in HaCat cells significantly repressed cell proliferation. However knockdown of Klf5 also blocked the TGF-β induced growth inhibition. Reciprocally, overexpression of Klf5 resulted in increased proliferation but also sensitized cells to TGF-β mediated growth inhibition. Thus Klf5 was converted from a pro-proliferative factor into an anti-proliferative factor upon TGF-β signaling. Mechanistically acetylation of Klf5 altered its binding to the p15 (CDKN2B) promoter, a bona fide target of TGF-β. Thus this study also reinforces the idea that protein modification of Klf5 can alter its function. It also highlights another level of complexity. In contrast to the effect of SUMOylation, acetylation appears to primarily affect the DNA-binding ability of Klf5. Additionally, these effects are likely to be promoter-specific since a previous study reported that the acetylation state of Klf5 does not alter its binding to the Klf5 binding sequence in EMSA [80].

Similar to Klf5, the Klf4 protein is also acetylated on residues Lys-225 and Lysine-229 by p300. The acetylation sites were required for the induction of the endogenous p21^cip1/waf1^ and Intestinal alkaline phosphatase (IAP) as well as for inhibition of proliferation [79]. However, mutation of the acetylation site did not affect the repressive function of Klf4 on the *CyclinB1* promoter in the same study.

## 6. ROLE IN TUMOUR DEVELOPMENT

Given that Klf4 and Klf5 have context dependent roles, it is not surprising that Klf4 and Klf5 possess both tumor suppressor and oncogenic functions. Klf4 is downregulated in many human cancers including colorectal [84], gastric [85, 86], bladder [87] and adult T cell leukemia [88]. In the pancreatic cancer cell lines, the Klf4 expression is associated with increased doubling time i.e. slower growth [15]. Klf4 is also downregulated in several types of B-lineage lymphomas and leukemias relative to normal tissue. Overexpression of Klf4 also suppressed the transformation of pre-B cells by ABL oncogenes [89]. Although these observations clearly define Klf4 as a tumor suppressor, it can also function as an oncogene. Schoenhals *et al.* [90] in their attempt to determine the expression of the embryonic stem cell markers in cancers, found an association of Klf4 overexpression with acute lymphoblastic leukemia, hairy cell leukemia and multiple myeloma. Additionally, Klf4 is overexpressed in oral squamous cell carcinoma and primary ductal carcinomas of the breast [30, 91].

The complexity does not end here since Klf4 can function either as an oncogene or tumor suppressor in the same tumor type. For instance, *in vitro* experiments using Estrogen receptor (ER) α positive breast cancer cell line MCF7 have revealed that estrogen dependent cell-growth was significantly enhanced by knockdown of Klf4 [92]. This would be consistent with the growth inhibitory function of Klf4. Earlier studies have however shown that 70% of breast cancers have elevated expression of Klf4 and that increased nuclear staining of Klf4 is associated with a more aggressive phenotype [91]. The inconsistencies between the two studies might reflect alterations in genetic context.

Mouse models have further highlighted the tumour suppressor versus oncogenic function of Klf4. Haploinsufficiency of Klf4 promotes adenomatous polyposis coli (APC)-dependent intestinal tumorigenesis [93]. In this study the *Apc^Min/+^* mouse model which is a well established model of intestinal tumorigenesis was used. The tumor burden in *Klf4^+/-^/Apc^Min/+^* mice was significantly increased compared to the *Apc^Min/+^* mice. In the skin however, overexpression of Klf4 resulted in the development of squamous cell carcinoma [30].

Klf5 can also function as either a tumour suppressor or an oncogene. Its expression is downregulated in several human cancers including those of the breast [24], prostate [80] and esophagus [27]. Moreover, the degradation of Klf5 was enhanced in cancer cell lines from breast and prostate as compared to the non-neoplastic cells [76]. It was also shown that Klf5 overexpression in non-transformed intestinal cell lines promoted their growth while Klf5 overexpression in transformed cell lines led to growth inhibition. Further Ras-mediated transformation of intestinal epithelial cells led to altered growth related properties of Klf5 [19]. Namely, Klf5 overexpression inhibited growth of Ras-transformed intestinal epithelial cells. Although these results are consistent with the observations made in breast [24], prostate [25] and esophageal [27] cancer cell lines, data contradicting a Klf5 growth inhibitory role in transformed cells is also available [20].

Regarding the oncogenic potential of Klf5, it has been shown to mediate the transforming activity of oncogenic H-Ras in NIH-3T3 cells [94]. Klf5 is highly expressed in human primary colorectal cancers exhibiting K-Ras mutations [20]. Its expression is also high in gastric cancer patients [95]. Furthermore, Klf5 expression has been suggested as a prognostic factor for overall survival in patients with sporadic breast cancer, with higher Klf5 expression correlating with shorter disease free survival and poorer overall survival [96]. Klf5 also promotes cell proliferation and tumorigenesis of the TSU-Pr1 human bladder cancer cell line [97] and Klf5 overexpression may be linked to salivary gland tumors [98]. Further in the mouse model of intestinal tumorigenesis, haploinsufficiency of KLF5 rescues the tumor initiating effect of the *Apc^Min^* mutation in the intestine [99].

## 7. UNITY IN DIVERSITY

As discussed, Klf4 and Klf5 control diverse functions in which they might antagonize or co-operate with each other. The data generated from the available mouse models shows that the consequence of Klf4 or Klf5 alteration is very often a change in lineage commitment/ differentiation programmes (discussed in section 3). It is possible that the diverse functions of Klf4 and Klf5 reflect their main function as ´priming-factors` or ´Fate-keepers` for signal induced differentiation/ lineage commitment. It is tempting to speculate that a sinusoidal wave of expression of these factors along the axis of lineage hierarchy may determine the sequential appearance of different cell types. This idea is represented in Fig. (**2**). In this regard it is noteworthy that Klf5 expression is high in the ESC but downregulated in pluripotent cell-lines generated from the epiblast of mouse embryos (EpiSC) [48]. The EpiSC have a more restricted pluripotency than embryonic stem cells. Further, Klf5 is also downregulated in the adult bulge hair follicle stem cells. This downregulation is however not associated with quiescence (see section 4). Interestingly, although Klf5 is downregulated in the bulge stem cells of the hair follicles, its expression goes up in the hair germ cells. The latter defines a cluster of cells in the hair follicle in a transitional state between the bulge stem cells and the matrix transit amplifying cells with a high proliferative potential [52]. These observations would be consistent with the model depicted in Fig. (**2**). How can Klf4/5 accomplish such a task? Unlike several transcription factors that are tightly coupled to specific signaling pathways like Gli or smads, Klf4 and Klf5 are not restricted to a single signaling pathway. It is possible that these factors are functioning as flagposts on the genome, priming it for signal-mediated activation and lineage commitment. ChIP on ChIP experiments would go far in addressing this issue. Future experiments elucidating the molecular mechanisms behind Klf4/5 functions will be instrumental in determining the validity of this model.

## 8. CONCLUDING REMARKS

Regenerative medicine has become very attractive since the observation that it is possible to generate patient specific iPS cells. An understanding of the basic mechanisms as to how cell fates are regulated is expected to provide novel means of manipulating this process. The therapeutic potentials of such a manipulation are far reaching. In this regard, Klf family members provide an excellent target for investigation. 

## Figures and Tables

**Fig. (1):**
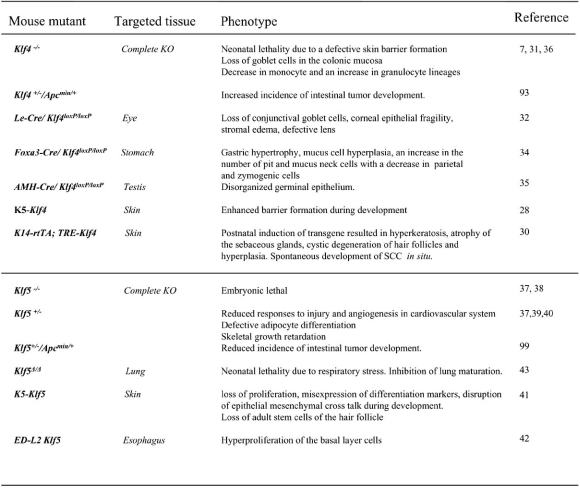
A summary of phenotypes arising in mice due to deletion or ectopic expression of Klf4 and Klf5.

**Fig. (2):**
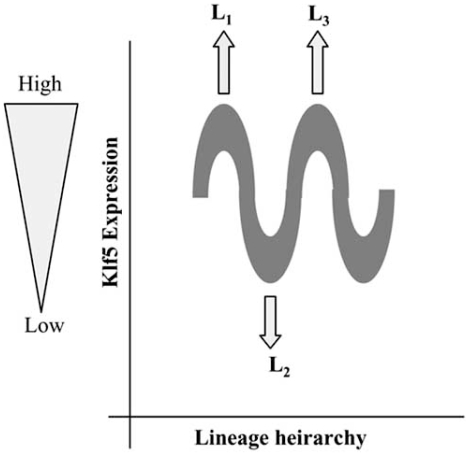
Model depicting the Fate-keeper function of Klf5. A sinusoidal wave of expression of Klf5 along the axis of lineage hierarchy may determine the sequential appearance of different lineages/cell types. These are represented in the figure as L1, L2 and L3.
